# Prominent Enlargement of the Facial Nerve Canal in a Case of a Persistent Stapedial Artery

**DOI:** 10.5334/jbsr.3361

**Published:** 2024-01-17

**Authors:** Alexandra Libeert, Stefan Delrue, Marc Lemmerling

**Affiliations:** 1 AZ Sint Jan Brugge-Oostende, Brugge, BE; 2 AZ Sint-Lucas Gent, BE; 3 Universitair Ziekenhuis Antwerpen (UZA), BE

**Keywords:** facial nerve canal, foramen spinosum, persistent stapedial artery, conductive hearing loss, computed tomography

## Abstract

In this case report, we present a 23-year-old patient with complaints of a recurrent plop sound in the left ear. Non-contrast CT and MR imaging revealed an enlargement of the tympanic segment of the facial canal due to venous congestion. A persistent stapedial artery (PSA) and absent foramen spinosum were identified as concomitant factors. This unique combination of anomalies has not been previously reported and knowledge about this condition is essential to avoid unnecessary surgery.

**Teaching Point:**

A persistent stapedial artery may be associated with enlargement of the tympanic facial nerve canal due to venous congestion.

## Introduction

The facial canal, also known as the fallopian canal, is a Z-shaped canal located within the temporal bone. Enlargement of the facial canal can be a cause for medical concern, potentially requiring surgery to address the underlying disorder. Herein, we report a rare case of fallopian canal enlargement based on venous congestion in a patient with concomitant foramen spinosum atresia and a persistent stapedial artery (PSA).

## Case Report

**Figure 1:**
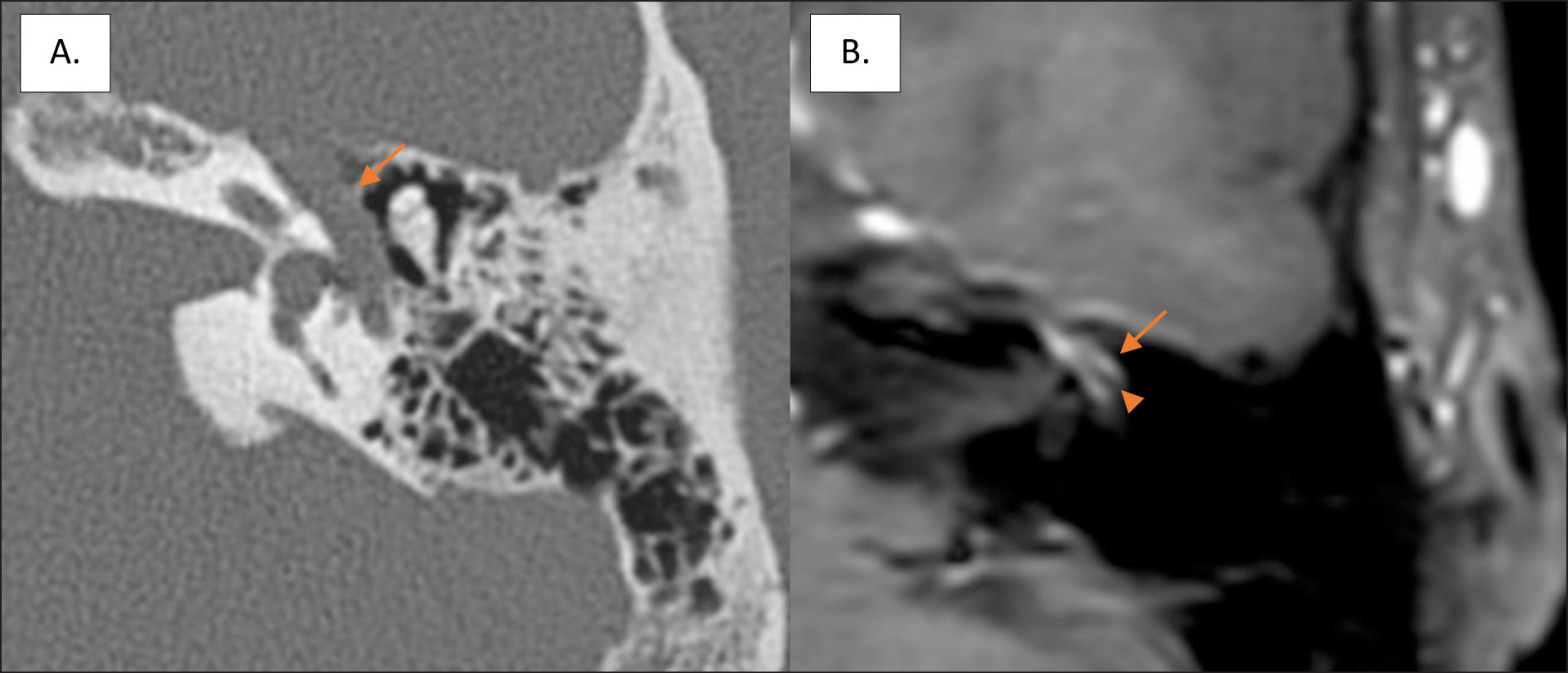
Enlargement of the tympanic segment of the fallopian canal (arrow) on axial CT scan of the left temporal bone **(A)** and intense contrast-enhancement (arrow) surrounding the facial nerve (arrowhead) on axial gadolinium-enhanced T1-weighted imaging **(B)**.

**Figure 2:**
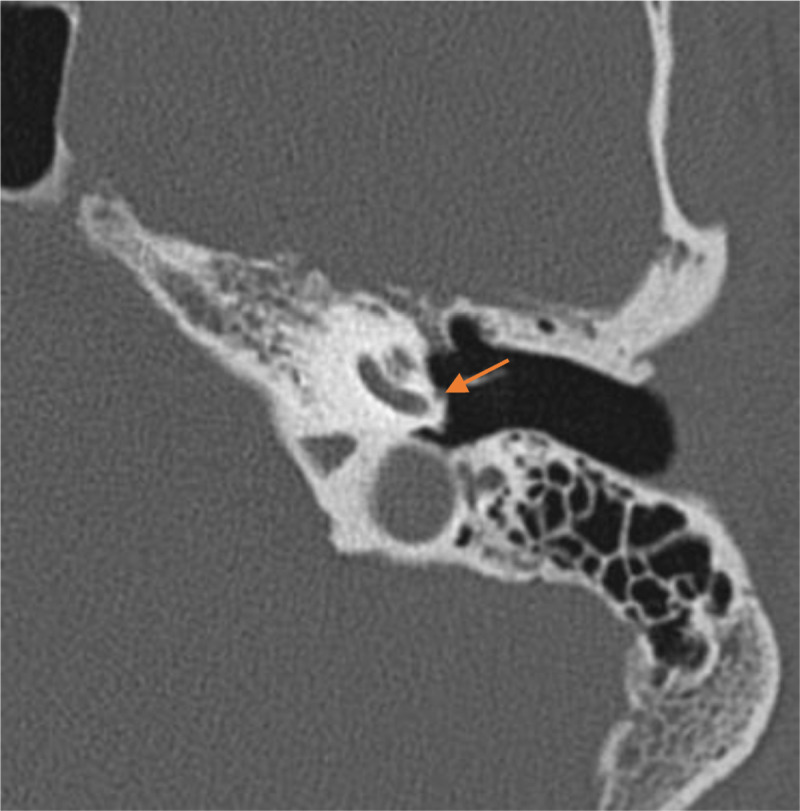
Axial CT scan showing the PSA on the cochlear promontory.

**Figure 3:**
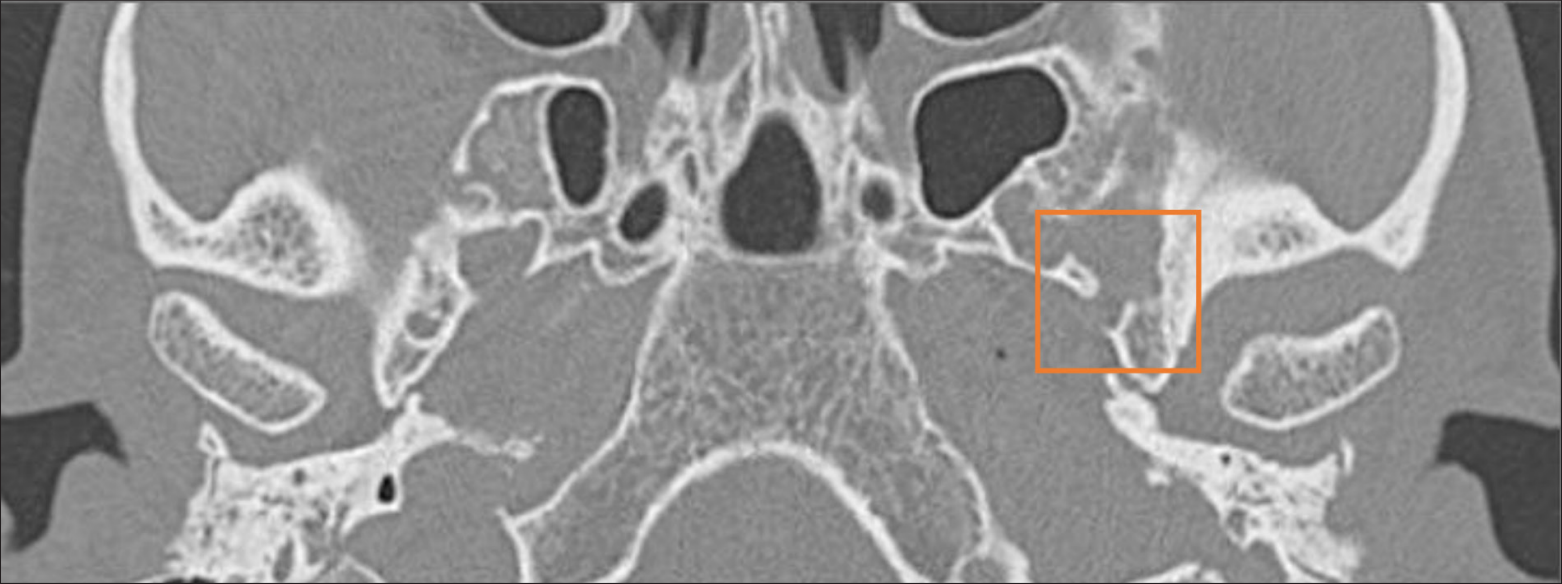
Axial CT scan showing the absence of the left foramen spinosum.

A 23-year-old male patient presented at the otorhinolaryngology department with complaints of a recurrent plop sound in the left ear. Physical examination was normal. Pure tone audiometry showed a slight conductive hearing loss at the low frequencies. Non-contrast computed tomography (CT) of the left temporal bone was performed, showing an enlargement of the tympanic segment of the fallopian canal (Figure 1A). A closer look to the scan revealed a small vessel running over the middle ear promontory which led to the diagnosis of a PSA (Figure 2). In addition, CT demonstrated an agenesis of the left foramen spinosum (Figure 3). A magnetic resonance (MR) scan was scheduled for further evaluation. Intense contrast enhancement surrounding the tympanic segment of the facial nerve was noted on gadolinium-enhanced spin echo based T1-weighted images, indicating congestion of the venous plexus surrounding the embedded facial nerve (Figure 1B). Right temporal bone imaging was normal. No treatment was scheduled.

## Discussion

The blood supply to the facial nerve is provided by different arteries, depending on the nerve segment. The tympanic segment of the facial nerve is most commonly supplied by the petrosal artery, which arises from the middle meningeal artery after passing through the foramen spinosum. In this case, an alternative blood supply through a PSA was observed. The stapedial artery usually regresses by ten weeks of gestation. Persistence of the artery is a relatively rare condition, occurring in approximately 0.02–0.48% of the population [1]. The PSA branches off from the petrous internal carotid artery, runs over the cochlear promontory, passes through the obturator foramen of the stapes and finally enters the tympanic segment of the fallopian canal. The typical feature of a PSA on CT imaging is a small soft-tissue prominence along the cochlear promontory (Figure 2). This condition frequently presents with an accompanying absence of the ipsilateral foramen spinosum, which was observed in our patient.

The most striking element in this case is the prominent enlargement of the entire tympanic segment of the facial canal, which overshadows the tiny artery on the promontory. Similar enlargements of the fallopian canal on CT can be found in various conditions, such as facial nerve schwannomas, neurofibromas and haemangiomas.

In this case, the widening of the canal is caused by venous congestion of the plexus surrounding the facial nerve. This is confirmed by the prominent contrast enhancement around a normal facial nerve on MR imaging (Figure 1B). On the basis of literature data, we can confidently suppose that a vein rather than an artery causes the canal enlargement [2,3].

A venous cause for facial canal enlargement has been nicely described on both histopathology and CT by Moonis et al. [2]. In another case report by Kim et al., in which similar findings on MR to our case were described, venous engorgement around the facial nerve was observed intraoperatively [3]. In these cases, there was no PSA. In normal physiology, the middle meningeal vein plays an important role in the venous drainage of the tympanic segment of the facial nerve canal. Like its arterial counterpart this vein typically passes through the foramen spinosum. Accordingly, absence of the foramen spinosum—which is often the case in a PSA—by definition results in different venous outflow. In our case, we hypothesize that the anomalous middle meningeal vein leads to congestion of the plexus surrounding the tympanic segment of the facial nerve. The combination of a PSA, absent foramen spinosum and a venous cause of enlargement of the facial nerve canal may seem logical, but it has never been described. Further research is necessary to confirm this theory.

The conductive hearing loss observed in our patient can be caused by the PSA and/or by fallopian canal enlargement, as these conditions may both limit the movement of the stapes. As there were only limited complaints, no middle ear exploration was scheduled.

## Conclusion

A persistent stapedial artery and the concurrent absence of the foramen spinosum may be associated with fallopian canal enlargement, caused by venous congestion. Knowledge of this association is important to prevent unnecessary interventions.
